# Effectiveness and Safety of a Novel Catheter‐Over‐Needle Assembly for Postoperative Analgesia in Orthopedic Limb Surgery: A Multicenter, Randomized, Single‐Blind, Active‐Controlled Noninferiority Trial

**DOI:** 10.1155/prm/7529711

**Published:** 2026-04-29

**Authors:** Bin Yu, Hao Fang, Tao Chang, Shukun Fu, Shuqi Xie, Zitong Zhao, Nu Zhang, Hongcheng Zhao, Xiaodan Cao, Xiyuan Shen

**Affiliations:** ^1^ Department of Anesthesiology and Pain Rehabilitation, Cardiopulmonary and Critical Care Rehabilitation Center, Shanghai Yangzhi Rehabilitation Hospital (Shanghai Sunshine Rehabilitation Center), School of Medicine, Tongji University, Shanghai, China, tongji.edu.cn; ^2^ Department of Anesthesiology, Zhongshan Hospital, Fudan University, Shanghai, China, fudan.edu.cn; ^3^ Department of Anesthesiology, Shanghai Geriatric Medical Center, Shanghai, China; ^4^ Department of Anesthesiology, Tenth People’s Hospital of Tongji University, Shanghai, China, shdsyy.com.cn; ^5^ Department of Anesthesiology, Shanghai Tongji Hospital, School of Medicine, Tongji University, Shanghai, China, tongji.edu.cn; ^6^ Department of Anesthesiology, Minhang Hospital, Fudan University, Shanghai, China, fudan.edu.cn

**Keywords:** continuous peripheral nerve block analgesia, continuous peripheral nerve block assembly, limb fracture surgery, nerve block catheters, postoperative analgesia

## Abstract

**Purpose:**

Indwelling nerve catheters are commonly used for postoperative analgesia following limb fracture surgeries. This multicenter, randomized, single‐blind, active‐controlled parallel trial aimed to compare the novel *Tuoren* catheter‐over‐needle assembly with the *Contiplex D* catheter‐through‐needle assembly for postoperative pain management.

**Methods:**

The trial was conducted at three centers in China. Patients undergoing elective limb fracture surgery were randomly assigned (1:1) to either the *Tuoren* group or the *Contiplex D* group for postoperative analgesia. The primary outcome was the noninferiority of the *Tuoren* assembly compared to that of the *Contiplex D* assembly in terms of the 24‐h postoperative analgesia effectiveness rate. Secondary outcomes were patient and surgeon satisfaction, safety, and the time required for puncture and catheter insertion. The rate of local anesthetic leakage and the incidence of catheter kinking and dislodgement during analgesia were also evaluated.

**Results:**

Between June 2019 and June 2021, 232 patients were randomized. The 24‐h postoperative analgesia effectiveness rate was 99.1% in both groups. After adjusting for surgery types and centers, the difference was 0.06% [−2.36%, 2.49%] with a 95% CI. The lower boundary was above the noninferiority limit of −10%. The insertion time for the *Tuoren* assembly was significantly shorter compared to that of the *Contiplex D* assembly. Other outcomes did not show statistical differences.

**Conclusions:**

The novel *Tuoren* assembly was noninferior to the *Contiplex D* catheter‐through‐needle assembly in terms of analgesic effectiveness and safety.

**Trial Registration:** Chinese Registry of Clinical Trials: ChiCTR1900022980

## 1. Introduction

Ultrasound‐guided peripheral nerve block is an effective method for providing postoperative analgesia in patients undergoing surgery for limb fractures and has become widely acceptable for both anesthesiologists and patients [1, 2]. To achieve effective postoperative pain relief, continuous peripheral nerve block utilizing a perineural catheter is often required [3–5]. Traditionally, nerve blocks are performed using either a nerve stimulator or ultrasound, with the *Contiplex D* catheter‐through‐needle approach. In this method, the catheter is advanced through the needle and beyond it. However, the outer diameter of the catheter is smaller than that of the skin puncture needle, leaving a space between the catheter and skin. This gap increases the risk of local anesthetic leakage at the insertion site and can also lead to catheter dislodgement, which compromises the effectiveness of the analgesia [6]. Additionally, the approach can be complicated, particularly when guided by a nerve stimulator. This not only prolongs the time needed for preoperative anesthesia but also causes discomfort for the surgeon and increases the overall length of the procedure.

Tsui et al. developed a catheter‐over‐needle approach for continuous peripheral nerve block by fitting the catheter over the needle, thus removing the gap between the catheter and skin [6, 7]. This approach is enabled by real‐time ultrasound guidance of catheter positioning [8–10]. Clinical trials have demonstrated that this method could provide effective analgesia while lessening the risks of catheter dislodgement and local anesthetic leakage [11, 12]. However, few trials have compared the effectiveness of the catheter‐over‐needle assembly *versus* the catheter‐through‐needle assembly using postoperative analgesia as the primary efficacy endpoint in a noninferiority design.

In recent years, continuous nerve block technology has been increasingly used for postoperative pain management in China. However, the widespread adoption of this technique has been limited by the lack of domestically available indwelling nerve block assembly, as well as the high cost of imported devices, which also suffer from design flaws, such as leakage at the puncture site, infection, and catheter displacement. Despite its clear benefits, continuous nerve block has not been widely implemented in clinical practice.

To address these issues, we designed a single‐use peripheral continuous nerve block assembly (Patent No.: ZL 2012 1 0471509.4). This novel catheter‐over‐needle assembly primarily consists of a 22‐gauge needle and a 90‐mm external indwelling catheter, and the needle features a 30°beveled tip (Supporting Figure 1A). This assembly features an innovative needle‐outside catheter design, which eliminates the puncture site leakage problem and allows for a single‐step insertion with a high success rate. The assembly is easy to use and has demonstrated excellent clinical outcomes in brachial plexus, femoral, and sciatic nerve blocks for anesthesia and postoperative analgesia. The catheter‐over‐needle assembly has received approval from China’s National Medical Products Administration (NMPA) and is manufactured by *Tuoren Medical Device Co.*

Tsui et al. demonstrated in animal studies that catheter‐over‐needle techniques offer potential advantages in leak pressure and catheter dislocation resistance [6, 7]. Consistently, our previous study first established the feasibility of the catheter‐over‐needle assembly for upper limb surgery. A subsequent preliminary comparison with the *Contiplex A* system (B. Braun, Germany) for post‐total knee arthroplasty analgesia suggested potential advantages of our assembly in terms of easier catheter insertion and reduced local anesthetic leakage [13, 14]. In this multicenter trial, we enrolled a larger sample of patients undergoing orthopedic limb surgeries to evaluate the effectiveness and safety of the *Tuoren* single‐use peripheral continuous nerve block assembly against the *Contiplex D* assembly (B. Braun, Germany). This study will further validate efficacy of the *Tuoren* assembly in postoperative pain management when used for continuous modified interscalene brachial plexus block and continuous popliteal sciatic nerve block for postoperative pain relief in a randomized controlled trial. The outcomes will include pain relief, catheter insertion time, and the incidence of complications such as abnormal local sensation and movement during catheter insertion and retention, puncture site leakage, catheter kinking, dislodgement, infection, and other adverse events (AEs).

## 2. Methods

### 2.1. Ethics

The trial adhered to the Declaration of Helsinki and Good Clinical Practice guidelines. The study protocol was approved by the Institutional Review Board of Shanghai Tongji Hospital (2017‐426, 2017‐426‐GZ‐181031 and 2017‐426‐XZ‐190521). All participants provided written informed consent prior to enrollment. The trial is registered with https://www.chictr.org.cn (Registration number, registration date: May 6th, 2019). A 10‐mL blood sample will be collected per patient for testing: liver function (ALT, AST, TBIL), renal function (creatinine), bleeding risk (PLT/coagulation), fasting glucose, and pregnancy (β‐hCG). All samples will be destroyed after use.

### 2.2. Study Design and Participants

This multicenter, randomized, single‐blind, active‐controlled parallel trial enrolled patients aged between 18 and 70 years undergoing elective surgery for clavicle, humerus, malleolus, or calcaneal fractures at 2‐day orthopedics units at the Shanghai Tongji Hospital of Tongji University, Minhang Hospital of Fudan University, and 10th People’s Hospital of Tongji University. American Society of Anesthesiologists (ASA) I, II, or III patients were eligible if they had an expected postoperative analgesia duration > 24 h and were capable of operating patient‐controlled analgesia (PCA) equipment. The eligibility criteria are detailed in the Supporting Table 1.

### 2.3. Study Device

The Tuoren catheter was 80 mm long (20G‐1.1 × 80 mm), and the disposable needle for continuous nerve block had a 30°beveled needle point (Patent No.: ZL 2012 1 0471509.4, Supporting Figure 1A). It received approval from the National Medical Products Administration (NMPA) of China. The approval number is 20233081042, and the approval date is July 28, 2023.

The Contiplex catheter was 20G in diameter and 1000 mm long (0.85 × 1000 mm), and the needle was 18G in diameter and 80 mm long (1.3 × 80 mm) with a 15° beveled needle point.

### 2.4. Randomization and Masking

Participants were randomized 1:1 to undergo nerve block with the *Tuoren* catheter‐over‐needle assembly (*Tuoren Medical Device Co.*, Changyuan, Henan, China) or the *Contiplex D* catheter‐through‐needle assembly (B. Braun, Melsungen, AG, Germany). Randomization used a central computerized system, with permuted blocks of different sizes, and was stratified for surgical types (upper limb *vs.* lower limb) and centers. Only patients were unaware of group allocation. All efficacy measures except physician satisfaction were assessed by a blinded independent Endpoint Evaluation Committee.

### 2.5. Continuous Nerve Block

Patients with clavicle or humerus fractures were placed supine with heads rotated toward the nonoperative side. They underwent ultrasound‐guided continuous interscalene brachial plexus nerve block by a modified Winne’s method using a short axis out‐of‐plane technique [15]. A linear array transducer (SonoSite S II), with a depth setting of 2.7 cm, was placed between the anterior and middle scalene muscles 0.5 cm below the cricoid cartilage. After the brachial plexus roots were identified, the *Tuoren* (Supporting Figures 1B and 1C) or Contiplex assembly (Supporting Figures 1D and 1E) was inserted between the middle scalene muscle and the brachial plexus trunks and advanced 2–4 cm in a caudad and lateral direction toward the mid clavicle. When the needle bevel approached the brachial plexus roots, 20 mL of 1% lidocaine and 0.25% ropivacaine was injected and the *Tuoren* needle was immediately withdrawn while the indwelling catheter remained in place. Meanwhile, the Contiplex catheter was threaded 3 cm past the tip of the Tuohy needle. After negative aspiration, the needle was removed, and the catheter was secured.

Patients with malleolus or calcaneal fractures were placed in the lateral decubitus position and underwent continuous popliteal sciatic nerve block using a short‐axis out‐of‐plane technique. A linear array transducer, with a depth setting of 4.0 cm, was placed 7 cm above the popliteal fossa crease. After the bifurcation of the sciatic nerve was identified, an 8‐cm needle (*Tuoren* or Contiplex) was inserted; when the bevel reached the bifurcation, 15 mL of 1% lidocaine and 0.25% ropivacaine was injected (Supporting Figures 1F to 1I). The *Tuoren* needle was withdrawn, and after negative aspiration, the catheter was secured. Meanwhile, the Contiplex catheter was advanced 5 cm past the tip of the Tuohy needle in the perineural space. Written informed consent for publication was obtained from individuals depicted in the figure.

The insertion time was the interval between patients assuming the supine or lateral decubitus position and the time each catheter was secured. Nerve injuries, local movement disorders, puncture site infection, and bleeding were recorded.

### 2.6. Postoperative Analgesia and Assessment

At the end of surgery, the catheter was connected to the PCA pump for 48 h. Pain was evaluated using the visual analog scale (VAS) 24 h postoperatively. Intravenous parecoxib 40 mg was given to patients with VAS score > 3 at 24 h postoperatively. Subcutaneous morphine 0.1 mg/kg was provided to patients with VAS score > 3 within 15 min of receipt of parecoxib. Finally, 0.2% ropivacaine (5 mL, lockout 20 min, maximally 20 mL) was delivered *via* the indwelling catheter in patients with VAS score > 3 within 30 min of receipt of morphine. The PCA regimen was a bolus‐only mode with no background infusion. All centers utilized an identical PCA protocol and the same device model, ensuring consistency throughout the study.

Gross local anesthetic leakage and catheter kinking and slippage during analgesia were recorded. Patient and surgeon satisfaction with postoperative analgesia was assessed on day 2 postoperatively using a categorical 5‐point scale (0 = completely unsatisfied; 5 = completely satisfied). The overall satisfaction rate was the proportion of patients or surgeons with a satisfaction score of 4 and 5. The number, frequency, and severity of AEs were recorded. Significant AEs included, apart from serious AEs, those AEs requiring interventions.

The primary endpoint was the postoperative analgesia effectiveness rate. A VAS score of 3 or below was defined as indicative of effective postoperative analgesia, as it represents a state of “mild pain” that is generally well tolerated and permits comfortable patient mobilization. Secondary endpoints included patient and surgeon satisfaction score and the effective PCA pump usage rate, which was the total number of injections to that of attempts using the PCA pump × 100%.

### 2.7. Statistical Analysis

All analyses were prespecified. Sample size was calculated based on a postoperative analgesia effectiveness rate of 96.3% with Stimuplex (B. Braun) [16]. We assumed a postoperative analgesia effectiveness rate of 93.0% for each group, with an intergroup difference of 0%. With a prespecified noninferiority margin of 10%, a power of 90%, and a one‐sided alpha of 0.025. This margin was determined through comprehensive consideration of several factors: the anticipated high success rate of the intervention in clinical practice, historical margin ranges reported in previous noninferiority trials for interventions with similarly high efficacy, and consensus among the research team regarding the clinically acceptable boundary of efficacy loss [17, 18]. We acknowledge that this choice, while prespecified and clinically guided, involves inherent subjectivity. Noninferiority was established if the lower boundary of the 95% confidence interval (CI) exceeded −10% for the intergroup difference. It is assumed that the difference in effectiveness between the control group and the experimental group is 0. A minimal sample size of 103 participants was anticipated for each group. Assuming a 10% attrition rate, a population size of 116 was required per group. The following formulas were used:

(1) nT=nC=Z12−α/+Z1−β2PC1−PC+PT1−PTD−Δ2,

*P*_*T*_: sample proportion of the experimental group, *P*_*C*_: sample proportion of the control group, Δ: noninferiority margin., *α*: Type I error rate, *β*: Type II error rate, *n*_*T*_: sample size of the experimental group, *n*_*C*_: sample size of the control group, and *D*: expected difference between the two proportions.

Totally three subjects were excluded from FAS for the following protocol‐mandated reasons:

Intervention #35: Did not receive the device due to a change in surgical plan. Missing data will be excluded from the analysis.

Control #49: Voluntarily withdrew consent preoperatively and received no intervention.

Control #96: Was randomized but found to violate key inclusion criterion (BMI > 28) and did not receive the device.

Given that these exclusions are protocol‐driven and nonrandom, the primary endpoint analysis will be conducted using a worst‐case scenario imputation to assess the robustness of the results. Specifically, for the primary analysis of the 24‐h analgesic effectiveness rate:

For missing subjects in the intervention group (#35), the outcome will be imputed as treatment failure.

For missing subjects in the control group (#49 and #96), the outcome will be imputed as treatment success.

This conservative approach is designed to test whether the noninferiority conclusion holds under the most unfavorable assumptions regarding the missing data. The last observation carried forward (LOCF) method was considered inappropriate due to the absence of any postrandomization efficacy data for these subjects. No imputation was applied to secondary endpoints. The trial followed the intention‐to‐treat principle. Efficacy analyses were mainly based on the full analysis set (FAS) and the per‐protocol set (PPS). The PPS included patients who complied with the study protocol, demonstrated good compliance, and had complete data. Patient and surgeon satisfaction scores, the effective PCA pump usage rates, and the insertion time were compared using the Wilcoxon rank sum test. The rates of gross local anesthetic leakage, catheter kinking, and slippage during analgesia were compared using Fisher’s test. To quantify the impact of subjects who withdrew early from the trial on the conclusion, we performed a “tipping point” analysis. The tipping point analysis determined the threshold analgesic effectiveness rate required in the experimental group to maintain noninferiority, under the assumption that all missing data in the control group represented effective analgesia.

The safety set included all patients who had received at least one dose of the study medications and was mainly analyzed using descriptive statistics. The incidence of AEs was compared using the Fisher exact test.

Statistical analyses were done using SAS9.3 (The SAS Institute, Cary, NC, USA). All tests were two‐sided, and *p*‐values ≤ 0.05 were considered statistically significant.

## 3. Results

### 3.1. Patient Characteristics

**FIGURE 1:**
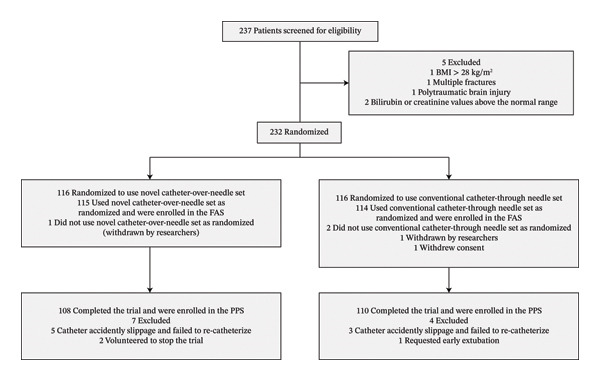
The consort flowchart. FAS: full analysis set. PPS: per protocol set.

Between June 2019 and June 2021, 237 patients were screened; 232 were eligible and randomized. The FAS included 229 patients (one patient in the *Tuoren* group and two patients in the *Contiplex D* group were withdrawn by researchers or due to patient consent withdrawal); 115 received continuous peripheral nerve block with the *Tuoren* catheter‐over‐needle assembly (1 missing) and 114 with the *Contiplex D* catheter‐through‐needle assembly. The PPS set included 108 patients in the *Tuoren* group and 110 in the *Contiplex D* group (5 and 3 patients were excluded because the catheter accidently slippage and failed to recatheterize in *Tuoren* and *Contiplex D* groups, respectively, 2 in the *Tuoren* group volunteered to stop the trial and 1 in the *Contiplex D* group requested early extubation; Figure 1). Their median age was 47 years and 59% were male. Seventy‐three patients (31.9%) had medial or lateral malleolus fractures, sixty‐five (28.4%) had clavicle fractures, and fifty‐three (23.1%) had humerus fractures. The two groups were comparable in baseline variables (Table 1). There were no sensory or motor abnormalities at the nerve innervation sites in either group of patients.

**TABLE 1:** Patient demographic and baseline characteristics‐FAS.

Variables	Catheter‐over‐needle (*n* = 115)	Catheter‐through‐needle (*n* = 114)
Age, years		
Median(range)	47 (37.70)	47 (36.58)
Sex, *n* (%)		
Male	69 (60.0)	68 (59.7)
Female	46 (40.0)	46 (40.4)
Ethnicities, *n* (%)		
Han	114 (99.1)	112 (98.4)
Other	1 (0.9)	2 (1.8)
BMI (kg/m^2^)		
Mean [SD]	23.5 (2.7)	23.8 (2.7)
Q1, Q3	21.5,25.7	21.8,25.4
ASA physical status		
I	32 (27.8)	42 (36.8)
II	82 (71.3)	72 (63.2)
III	1 (0.9)	0
Fracture sites		
Clavicle	32 (27.8)	33 (29.0)
Humerus	26 (22.6)	27 (23.7)
Medial or Lateral malleolus	38 (33.0)	35 (30.7)
Calcaneus	19	19
Center number		
01	59	60
02	34	33
03	22	21

*Note:* BMI is the weight in kilograms divided by the square of the height in meters.

Abbreviations: ASA, American Society of Anesthesiologists; BMI, body‐mass index.

### 3.2. Primary Outcome

In the FAS, out of 115 patients in the Tuoren catheter‐over‐needle assembly group (113 effective, 1 ineffective, 1 missing), 113 patients achieved effective analgesia at 24 h postoperatively. In the Contiplex D catheter‐through‐needle assembly group, 113 patients out of 114 patients (113 effective, 1 ineffective) had effective analgesia at 24 h postoperatively. The 24‐h postoperative analgesia effectiveness rate was 99.1% for the Tuoren catheter‐over‐needle assembly group versus 99.1% for the Contiplex D catheter‐through‐needle assembly group. The unadjusted intergroup difference was 0.0%, with all three statistical methods demonstrating no statistically significant difference between groups (Miettinen–Nurminen method 95% CI: −4.02%–4.02%, Newcombe–Wilson method 95% CI: −3.99%–3.99%; Newcombe–Wilson with continuity correction 95% CI: −4.70%–4.70%; Supporting Table 2).

Given the three cases with missing data, we analyzed these missing values using the worst‐case imputation method. The unadjusted intergroup difference in analgesic effectiveness rates was −0.88%, with all three statistical methods demonstrating no statistically significant difference between the groups (Miettinen–Nurminen method 95% CI: −5.04%–3.28%; Newcombe–Wilson method 95% CI: −5.01%–3.25%; Newcombe–Wilson method with continuity correction 95% CI: −5.73%–3.97%; Supporting Table 2).

To avoid the heterogeneity bias introduced by the patients who have undergone different surgeries in different centers, we adjusted the data for surgical types and centers. The difference was 0.06% (Mantel–Haenszel 95% CI ‐2.36%–2.49%; stratified Newcombe [Mantel–Haenszel weighted] 95% CI ‐6.95%–7.13%), 0.34% (Miettinen–Nurminen [inverse variance weighted] 95% CI ‐6.4%–7.1%), and 0.15% (stratified Newcombe [minimal risk weighted] −6.99%–7.26%) with the lower boundary of 95% CI above noninferiority limit‐10% (Table 2).

**TABLE 2:** Postoperative 24‐hour analgesic effectiveness rate difference (%) (test group—control group) and 95% confidence intervals after adjusting for covariates—FAS.

Covariate	Mantel–Haenszel		Stratified Newcombe (Mantel–Haenszel weight)		Stratified Newcombe (minimized risk weight)		Miettinen–Nurminen (inverse variance weight)	
	Rate difference (%)	95% CI	Rate difference (%)	95% CI	Rate difference (%)	95% CI	Rate difference (%)	95% CI
Surgery	−0.05	−2.46, 2.37	−0.05	−5.03, 4.93	−0.03	−5.06, 5.09	−0.01	−4.96, 4.94
Center	0.03	−2.39, 2.46	0.03	−5.60, 5.71	0.10	−5.57, 5.71	0.26	−4.91, 5.42
Surgery and Center	0.06	−2.36, 2.49	0.06	−6.95, 7.13	0.15	−6.99, 7.26	0.34	−6.40, 7.07

*Note:* 1. Surgery: Random stratification factor, binary variable (upper limb fracture, lower limb fracture); Center: Ternary variable, with three study centers. 2. One subject (device code 10, in the test group) had missing postoperative VAS data. This subject was excluded from the statistical analysis model by treating the missing data as nonevaluable when calculating the effectiveness rate.

A total of 108 patients in the *Tuoren* assembly group totally completed the trial and were enrolled in the PPS, and 108 (100%) patients achieved effective analgesia at 24 h postoperatively. In the *Contiplex D* catheter‐through‐needle assembly group, 108 (98.18%) out of 110 patients had effective analgesia at 24 h postoperatively in the PPS (Supporting Table 2 and 3). The lower boundary of the 95% CI exceeded −10%, establishing noninferiority of the *Tuoren* catheter‐over‐needle assembly.

In the PPS, there were 4 missing participants in the *Contiplex D* group and 7 in the *Tuoren* group. The “tipping point” analysis revealed that, even under the most unfavorable assumption, the noninferiority conclusion would only be reversed if the 24‐h analgesic effectiveness rate among the withdrawn patients in the experimental group fell below 20.5%, which was far lower than the probability of these patients having effective analgesia in this study. This suggests that the noninferiority conclusion is unlikely to be overturned.

### 3.3. Patient and Surgeon Satisfaction

Patients and doctors were asked to rate their satisfaction with the use of the indwelling catheter assembly on a scale of 1–5, with a score above 3 indicating overall satisfaction. In the FAS, the overall satisfaction rate of postoperative analgesia was 94.8% (4 patients gave a score of 3, 27 gave a score of 4, and 82 gave a score of 5) in the *Tuoren* catheter‐over‐needle assembly group versus 92.1% (7 patients gave a score of 3, 26 gave a score of 4, and 79 gave a score of 5) in the *Contiplex D* catheter‐through‐needle assembly group (*p* = 0.566, Wilcoxon rank sum test) (Figure 2(a)). In PPS, the overall satisfaction rate was 100% (2 gave a score of 4 and 106 gave a score of 5) in the *Tuoren* catheter‐over‐needle group *versus* 98.2% (2 gave a score of 3, 13 gave a score of 4, and 94 gave a score of 5) in the *Contiplex D* catheter‐through‐needle assembly group (*p* = 0.550, Wilcoxon rank sum test, data not shown).

FIGURE 2Patient and surgeon satisfaction with postoperative analgesia. (a) Patient 503 satisfaction with postoperative analgesia. (b) Surgeon satisfaction with postoperative analgesia. Patient and surgeon satisfaction with postoperative analgesia was assessed on day 2 postoperatively using a categorical 5‐point scale (0 = completely unsatisfied; 5 = completely satisfied).

**Figure:**
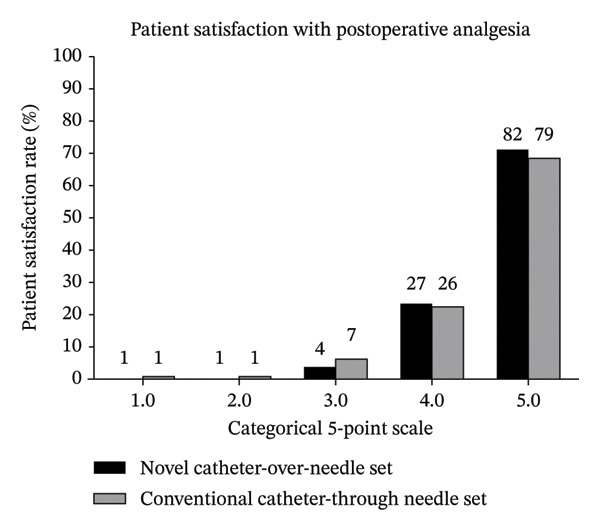
(a)

**Figure:**
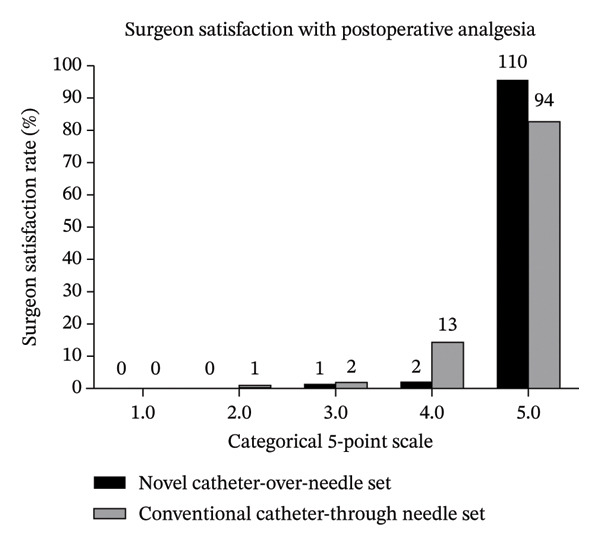
(b)

In FAS, the overall satisfaction rate in surgeons was 97.4% (1 gave a score of 3, 2 gave a score of 4, and 110 gave a score of 5) and 97.7% (2 gave a score of 3, 16 gave a score of 4, and 95 gave a score of 5) in *Tuoren*group and *Contiplex D* groups, respectively, with a significant intergroup difference (*p* < 0.01, Wilcoxon rank sum test) (Figure 2(b)). In PPS, the overall surgeon satisfaction rate is 100% (2 gave a score of 4 and 106 gave a score of 5) and 99.1% (2 gave a score of 3, 13 gave a score of 4, and 94 gave a score of 5), respectively (*p* < 0.01, Wilcoxon rank sum test, data not shown).

### 3.4. Safety Assessments

AEs occurred in 3 patients (2.61%) in the *Tuoren* catheter‐over‐needle assembly group and 5 (4.39%) in the *Contiplex D* catheter‐through‐needle assembly group. Allergic dermatitis, hypersensitivity, and eye disorder each occurred in 1 patient in the *Tuoren* catheter‐over‐needle assembly group. Fever occurred in 2 patients, and allergic dermatitis and constipation each occurred in 1 in the *Contiplex D* catheter‐through‐needle assembly group. Significant AEs occurred in 3 patients in the *Tuoren* catheter‐over‐needle assembly group and 2 in the *Contiplex D* catheter‐through‐needle assembly group. Serious AEs occurred in neither group. No AEs caused treatment interruptions or terminations. There were no treatment‐related AEs (Table 3).

**TABLE 3:** Adverse events in the two groups.

Adverse events (AEs)	Tuoren group, *n* (%)	Contiplex D group, *n* (%)
Total AEs	3 (2.6)	5 (4.4)
Serious AEs	0 (0.0)	0 (0.0)
Significant AEs	3 (2.6)	2 (1.8)
Adverse reactions	0 (0.0)	0 (0.0)
AEs leading to treatment interruptions	0 (0.0)	0 (0.0)
AEs leading to treatment terminations	0 (0.0)	0 (0.0)

*Any grade AEs*		
Fever	0 (0.0)	2 (1.8)
Allergic dermatitis^∗^	1 (0.9)	1 (0.9)
Headache	0 (0.0)	1 (0.9)
Hypersensitivity^∗^	1 (0.9)	0 (0.0)
Constipation^∗^	0 (0.0)	1 (0.9)
Eye discomfort^∗^	1 (0.9)	0 (0.0)

^∗^Significant AEs.

In both groups, there were cases where laboratory tests, electrocardiograms, chest X‐rays, vital signs, and physical examinations showed abnormalities postoperatively, despite being normal preoperatively, or where preoperative abnormalities persisted postoperatively. However, these postoperative abnormalities were deemed clinically insignificant (Supporting table 4 and 5).

No patients in each group had nerve injuries, local movement disorders, puncture site infection, or bleeding. These results indicated that there is no difference in safety between the two assemblies.

### 3.5. Nerve Block Procedural Characteristics

In the FAS, the *Tuoren* catheter‐over‐needle assembly group had an average insertion time of 3.86 ± 2.51 min with a median time of 3.0 min (interquartile range [IQR] 2.0–5.0), while the *Contiplex D* catheter‐through‐needle assembly group had an average insertion time of 6.61 ± 3.29 min with a median time of 5.0 min (IQR 5.0–8.0). The difference between the two groups was statistically significant (Wilcoxon rank sum test, *p* < 0.001; Table 4).

**TABLE 4:** Comparison of insertion time between the two groups—FAS/PPS.

	FAS		PPS	
	Tuoren	Contiplex D	Tuoren	Contiplex D
N (Nmiss)	115 (0)	114 (0)	108 (0)	110 (0)
Mean (SD)	3.86 (2.51)	6.61 (3.29)	3.91 (2.52)	6.65 (3.34)
Median	3.00	5.00	3.00	5.00
Q1, Q3	2.00, 5.00	5.00, 8.00	2.00, 5.00	5.00, 8.00
Min, Max	1.00, 12.00	1.00, 20.00	1.00, 12.00	1.00, 20.00
statistic	*Z* = 7.55		*Z* = 7.32	
*p* Value	< 0.0001		< 0.0001	

*Note:* Comparison between groups was performed using the Wilcoxon rank sum test.

The gross local anesthetic leakage occurred in 1 patient in the *Tuoren* catheter‐over‐needle assembly group and 2 in the *Contiplex D* catheter‐through‐needle assembly group at 24 h (Fisher exact test, *p* = 0.622). No patients in the *Tuoren* catheter‐over‐needle assembly group and 4 (3.5%) in the *Contiplex D* catheter‐through‐needle assembly group had gross leakage at 48 h (Fisher exact test, *p* = 0.122). Catheter kinking and slippage occurred in 2 and 4 patients in each group at 24 and 48 h, respectively (Table 5).

**TABLE 5:** Leakage rate and catheter kinking and slippage rate between the two groups.

Variables	Full analysis set (FAS)		Per protocol set (PPS)	
	Catheter‐over‐needle *n* (%)	Catheter‐through‐needle *n* (%)	Catheter‐over‐needle *n* (%)	Catheter‐through‐needle *n* (%)
*At 24 Hours*				
No Leakage	114 (99.13%)	112 (98.25%)	108 (100.00%)	108 (98.18%)
With Leakage	1 (0.87%)	2 (1.75%)	0 (0.00%)	2 (1.82%)
Total	115 (100.00%)	114 (100.00%)	108 (100.00%)	110 (100.00%)
*p*‐value	0.6217		0.4977	
No Kinking/Slip	113 (98.26%)	112 (98.25%)	108 (100.00%)	110 (100.00%)
With Kinking/Slip	2 (1.74%)	2 (1.75%)	0 (0.00%)	0 (0.00%)
Total	115 (100.00%)	114 (100.00%)	108 (100.00%)	110 (100.00%)
*p*‐value	1.0000		1.0000	

*At 48 Hours*				
No Leakage	113 (100.00%)	110 (96.49%)	108 (100.00%)	106 (96.36%)
With Leakage	0 (0.00%)	4 (3.51%)	0 (0.00%)	4 (3.64%)
Total	113 (100.00%)	114 (100.00%)	108 (100.00%)	110 (100.00%)
*p*‐value	0.1217		0.1216	
No Kinking/Slip	109 (96.46%)	110 (96.49%)	108 (100.00%)	110 (100.00%)
With Kinking/Slip	4 (3.54%)	4 (3.51%)	0 (0.00%)	0 (0.00%)
Total	113 (100.00%)	114 (100.00%)	108 (100.00%)	110 (100.00%)
*p*‐value	1.0000		1.0000	

*Note:* Fisher’s exact test was used for intergroup comparisons. Catheter kinking was indicated by an inability to inject fluids, and slippage was defined as unintentional dislodgment requiring replacement. The occurrence of either kinking or slippage was recorded as an event.

## 4. Discussion

The current multicenter study was conducted to expand the sample size to further validate the efficacy and safety of the *Tuoren* catheter‐over‐needle assembly based on preliminary research [13, 14]. The results demonstrated that the novel *Tuoren* catheter‐over‐needle assembly was noninferior to the conventional *Contiplex D* catheter‐through‐needle assembly in terms of 24‐h postoperative analgesic efficacy, a conclusion that remained robust under multiple sensitivity analyses. Furthermore, the new assembly exhibited a shorter insertion time and a lower rate of gross local anesthetic leakage while maintaining a comparable safety profile. Our findings are consistent with earlier studies showing that continuous popliteal sciatic nerve block exerts a durable analgesic effect lasting approximately 15 h postoperatively and reduces opioid consumption and the associated complications [19]. These findings validate the results of preliminary research and provide high‐level evidence to support the clinical application of this technique for postoperative analgesia following orthopedic limb surgery.

We set the noninferiority margin of −10% in the current study, which was determined based on the following considerations: First, the expected block effectiveness in our study population was approximately 93%. According to preliminary data and clinical experience, a margin of −10% implies a clinically acceptable lower bound of 83% effectiveness for the new intervention, as any potential increase in analgesic failure would be small in absolute magnitude and could be promptly and safely managed using conventional rescue analgesia protocols. Second, compared with precedents in regional anesthesia studies, which commonly employ noninferiority margins of 15%–20% (e.g., Dossi et al. used a margin of 20% for a nerve block with an expected efficacy of ∼90% in breast cancer surgery) [17, 18], our margin of −10% may be considered relatively more stringent.

We fully acknowledge that the selection of the noninferiority margin in this study involves a degree of subjectivity as it was not derived from a formal, prospectively defined minimal clinically important difference (MCID) specific to this clinical context as it was not derived from a formal, prospectively defined MCID specific to this clinical context. We explicitly recognize this as a methodological limitation of our work.

However, multiple lines of evidence indicate that this limitation does not undermine the reliability of the core conclusions. First, to rigorously address potential concerns about the robustness of our findings under these conditions, we employed multiple prespecified statistical methodologies that differ in their handling of binomial data and stratification approaches. Notably, analyses were conducted on both the FAS and the PPS. Critically, all methods converged to the same conclusion, confirming the noninferiority of the experimental intervention. The consistency across these diverse approaches significantly strengthens the credibility of the finding, ruling out the possibility that it is merely an artifact of a single underpowered test in a high‐success setting. Second, sensitivity analyses further confirmed the robustness of this conclusion, showing that the lower bound of the confidence interval for the risk difference remained well above −10%. Finally, in addressing the methodological challenge posed by the high efficacy rates approaching a ceiling effect (∼99.1%) in both groups, we assert that this statistical phenomenon does not diminish the clinical observation that both treatments are, in fact, overwhelmingly effective. The nearly identical point estimates themselves constitute strong prima facie evidence of clinical equivalence.

Furthermore, it should be noted that our primary outcome measure represents only one component of a comprehensive clinical evaluation. In the context of a pronounced ceiling effect, with both groups demonstrating a near‐maximal effectiveness (∼99.1%), an overly stringent margin focused solely on efficacy might preclude a meaningful assessment of other critical tradeoffs, such as procedure time, risk of local anesthetic leakage, and safety profile. Continuous peripheral nerve block provides effective analgesia beyond the duration of a single injection of local anesthetics [20, 21] whereby patients experience less severe pain and consume less opioids. Nearly all our patients receiving *Tuoren* catheter‐over‐needle assembly attained effective analgesia 24 h postoperatively. Moreover, the *Tuoren* catheter‐over‐needle assembly was safe overall and demonstrated comparable rates of AEs to the *Contiplex D* catheter‐through‐needle assembly. Meanwhile, it had a shorter insertion time and a lower rate of gross local anesthetic leakage *versus Contiplex D catheter-through*‐needle assembly. Our catheter‐over‐needle assembly provides a novel safe and effective analgesic method for the surgical treatment of limb fractures noninferior to currently available conventional methods.

Three cases were excluded from FAS for nonrandomized reasons. To conservatively assess their potential influence, we applied a worst‐case imputation method. The results demonstrated that the noninferiority conclusion remained valid. Notably, in this study, we successfully collected primary outcome data for all cases in the FAS and conducted analyses on both the FAS and the PPS. Additionally, to quantify the impact of subjects who withdrew early from the trial on the conclusion, we performed a “tipping point” analysis. This analysis revealed that, even under the most unfavorable assumption, the noninferiority conclusion would only be reversed if the 24‐h analgesic effectiveness rate among the withdrawn patients in the experimental group fell below 20.5%. Reviewing the data, all 7 withdrawals in the experimental group were attributed to mechanical catheter issues, with none recorded as “analgesic failure,” and several reports noted patients were “pain‐free and comfortable” at the time of withdrawal. Therefore, clinical judgment suggests that the true probability of effective analgesia in these patients is substantially higher than this tipping point. This is consistent with the results obtained from the FAS. These results strongly demonstrate that the core conclusion of our study is highly robust to missing data.

In the trial, nerve block was guided by ultrasound instead of neurolocalization with a nerve stimulator. Ultrasound guidance can provide useful information about the appropriate depth of the brachial plexus or the sciatic nerve [22–24]. A Tuohy needle was reported to be associated with a false‐negative motor response rate, causing unnecessary needle manipulations, needle withdrawals, or reinsertions, thus prolonging procedural time [25]. We observed a notably shorter insertion time with the *Tuoren* catheter‐over‐needle assembly than the *Contiplex D* catheter‐through‐needle assembly (3.0 min *vs.* 5.0 min). In the latter, the Contiplex catheter was threaded 3–5 cm past the tip of the Tuohy needle, which added to insertion time, while in the former, the indwelling catheter was left in place upon withdrawal of the *Tuoren* needle. While this time saving may not represent an order‐of‐magnitude improvement, it occurs during a critical phase of anesthesia induction and surgical preparation. A saving of 2–3 min can translate into a faster onset of anesthetic effects, earlier positioning for surgery, and potentially contribute to an improved operating room turnover. This is particularly relevant given that our study population included elderly patients, who are more susceptible to hemodynamic fluctuations. A more efficient and stable induction phase provides a valuable time buffer for managing such potential instabilities, thereby enhancing the overall safety margin of the procedure.

Local anesthetic leakage and catheter dislodgement are common complications of nerve block with conventional catheter‐through‐needle methods. To lessen their occurrences, Tsui et al. developed a catheter‐over‐needle assembly and showed that the *Tuoren* catheter‐over‐needle assembly had a lower rate of gross local anesthetic leakage than the *Contiplex D* catheter‐through‐needle assembly (0.9% *vs.* 5.3%)^8^. Unlike the technique of in‐plane femoral perineural catheter insertion in their study, our assembly uses ultrasound‐guided short‐axis out‐of‐plane continuous interscalene brachial plexus nerve block and popliteal sciatic nerve block, which have the advantage of reducing peripheral nerve injury by allowing catheters to travel along the nerves under visualization. Meanwhile, a unique innovation was made in the structure of the needle to further prevent drug leakage by adding side holes to facilitate anesthesiologists to administer drugs while advancing the needle. Furthermore, the rate of catheter kinking and slippage is comparable in the two groups, which falls within the reported range (6%–15%) [26].

The catheter in the Tuoren assembly is constructed from fluorinated ethylene propylene (FEP, Teflon), chosen for its stiffness to facilitate smooth tissue penetration. In contrast, the catheter in the Contiplex D assembly is made of a softer, more flexible polyurethane blend. In our trial, we observed comparable rates of catheter kinking between the two groups. Although the stiff FEP material is theoretically more prone to kinking during sharp bending, no operator reported subjective difficulty in handling or positioning the Tuoren catheter. Anecdotal feedback from several operators suggested that the initial stiffness of the Tuoren catheter was advantageous for precise placement under ultrasound guidance, but care was taken to avoid acute angles at the skin entry point to prevent kinking. However, to further optimize the design, future iterations of catheter‐over‐needle assemblies could explore novel composite or copolymer materials that combine an initial rigidity for easy insertion with increased flexibility postplacement to reduce long‐term kinking risk. Incorporating radiopaque stripes or depth markers could also enhance visualization and placement accuracy under both ultrasound and fluoroscopy. Overall, the *Tuoren* catheter‐over‐needle assembly is safe and no new safety concerns emerged. Heart rate variabilities and cardiovascular instability were reported in patients receiving interscalene brachial plexus block [27]. In the trial, we observed no clinically meaningful heart rate variabilities and cardiovascular instability in patients receiving continuous nerve block, including continuous interscalene brachial plexus nerve block, with either *Tuoren* catheter‐over‐needle assembly or *Contiplex D* catheter‐through‐needle assembly. Adequate postoperative analgesia remains the primary goal of surgeons and contributes to patient satisfaction with surgical outcomes, and effective postoperative analgesia has been shown to enhance surgical recovery and improve patient satisfaction [28, 29]. Approximately 95% of our patients and 97% of the surgeons were either completely satisfied or satisfied with postoperative analgesia with the *Tuoren* catheter‐over‐needle assembly. Surgeon satisfaction is intrinsically linked to key practical outcomes such as ease of use and procedural success rate. An improvement in satisfaction likely reflects a more efficient and reliable workflow in the operating room.

In terms of pain relief efficacy following orthopedic surgery, our catheter‐over‐needle assembly proved to be noninferior to the *Contiplex D* catheter‐through needle assembly, providing robust evidence for its widespread application in actual clinical practice. The higher satisfaction levels among surgeons with this catheter‐over‐needle assembly also favors its further promotion in orthopedic surgeries for elderly patients. In conjunction with our innovatively developed continuous single‐limb regional nerve block anesthesia and postoperative pain relief techniques, it can facilitate enhanced postoperative recovery in elderly patients. Future research should investigate whether this assembly can effectively reduce postoperative complications in elderly patients due to anesthesia techniques, thereby broadening its clinical indications.

In this trial, only device‐related safety and efficacy indices and satisfaction among patients and surgeons were observed for the new catheter‐over‐needle assembly; an in‐depth study on postoperative complications will be conducted subsequently for the new assembly. Additionally, strict eligibility criteria were used in the study to ensure the reliability of the pain primary outcome, which, however, may limit the generalizability of the study findings (e.g., for subjects aged above 70 years).

This trial has several additional strengths. It was a multicenter, single‐blind, noninferiority randomized controlled study with a sufficiently powered sample size, relevant clinical outcomes were assessed, and potential confounders were addressed in the analysis. In addition, the 24‐h postoperative analgesic efficacy, patient analgesic pump compression efficacy, and 24‐h immediate efficacy were evaluated separately for pain level, which has the advantage of being sufficiently and comprehensively evaluated in the new catheter‐over‐needle assembly for postoperative analgesia.

## 5. Conclusions

Continuous peripheral nerve block using catheter techniques is frequently used for postoperative analgesia in orthopedic patients undergoing limb surgery. The conventional catheter‐through‐needle approach is complicated and uses an indwelling catheter, which incurs a risk of local anesthetic leakage at the catheter insertion site and catheter dislodgement. In this multicenter, randomized, controlled trial, we demonstrate that our catheter‐over‐needle assembly could achieve adequate postoperative analgesia in patients undergoing orthopedic limb surgery and causes no safety concerns. With ease of operation, this novel assembly offers adequate pain relief and improves both patient and surgeon satisfaction and should be explored for postoperative analgesia in other surgical settings.

## Author Contributions

Bin Yu and Shuqi Xie codesigned the study. Tao Chang, Xiaodan Cao, and Xiyuan Shen supervised the study’s quality. Bin Yu, Shuqi Xie, Hao Fang, Shukun Fu, Zitong Zhao, Nu Zhang, Hongcheng Zhao, Tao Chang, Xiaodan Cao, and Xiyuan Shen conducted the clinical trial. Bin Yu, Tao Chang, Xiaodan Cao, and Xiyuan Shen performed data cleaning and statistical analysis. Bin Yu, Shuqi Xie, and Hao Fang wrote the paper, and all authors reviewed the final manuscript. Bin Yu, Hao Fang, and Tao Chang contributed equally to this work and share first authorship.

## Funding

This trial was supported by Henan Tuoren Medical Device Co., Ltd., including provision of all Tuoren catheter assemblies used in the trial and financial grant for data collection and independent statistical analysis, the National Natural Science Foundation of China (Grant No. 82471242), the Science and Technology Commission of Shanghai Municipality (Grant No. 23Y11906500), the Shanghai Research Center of Rehabilitation Medicine (Top Priority Research Center of Shanghai) (Grant No. 2023ZZ02027), and the Shanghai Shenkang Hospital Development Center Municipal Hospital Diagnosis and Treatment Technology Promotion and Optimization Management Project (Grant No. SHDC12024147).

## Disclosure

The study protocol was independently developed and approved by the principal investigators. *Tuoren Medical Device Co.* was not involved in patient recruitment, data collection, statistical analysis, or the drafting of the manuscript. The independent statistician Ms. Wang Yuemei from the Statistics Department of the Chinese Naval Medical University had full access to the data and performed the analyses solely under the direction of the authors. The interpretation of results, manuscript preparation, and the decision to submit for publication were made exclusively by the authors.

## Ethics Statement

This study was approved by the Ethics Review Committee of Shanghai Tongji Hospital (2017‐426, 2017‐426‐GZ‐181031, and 2017‐426‐XZ‐190521). The approval date was May 21, 2019. The trial is registered with https://www.chictr.org.cn (Registration number:, registration date: May 6th, 2019).

## Consent

All patients provided written informed consent.

## Conflicts of Interest

Bin Yu is named as the inventor of the Tuoren assembly in this study (Patent No.: ZL 2012 1 0471509.4). The patent has been transferred to *Tuoren Medical Device Co., Ltd.* Other authors declare no conflicts of interest.

## Supporting Information

Supporting Table 1 outlines the inclusion criteria and exclusion criteria for the current clinical study.

Supporting Table 2 presents the 24‐h postoperative analgesia effectiveness rate comparison between two groups (Tuoren Group vs. Contiplex D Group) across different study centers, along with 95% confidence intervals (CIs) calculated using three different statistical methods.

Supporting Table 3 presents the 24‐h postoperative analgesia effectiveness rate comparison between the Tuoren Group and Contiplex D Group in the PPS, accounting for potential confounding factors (surgery type and study center). The analysis uses two statistical adjustment methods to provide robust estimates.

Supporting Table 4 presents safety data comparing Tuoren Group vs. Contiplex D Group across laboratory tests, electrocardiograms (ECGs), and chest X‐rays during the 48‐h treatment period and prescreening phase.

Supporting Table 5 compares vital signs (blood pressure, heart rate, oxygen saturation, and body temperature) between the Tuoren and Contiplex D groups at three time points (0 h, 24 h, and 48 h).

Supporting Figure 1 illustrates the design and clinical application of the patented Tuoren catheter‐over‐needle assembly in comparison to that of the Contiplex D catheter‐through‐needle assembly for upper and lower limb continuous nerve blocks.

## Supporting information


**Supporting Information** Additional supporting information can be found online in the Supporting Information section.

## Data Availability

Data are available on request due to privacy/ethical restrictions.
