# Supplementary material for: Effectiveness and Safety of a Novel Catheter‐Over‐Needle Assembly for Postoperative Analgesia in Orthopedic Limb Surgery: A Multicenter, Randomized, Single‐Blind, Active‐Controlled Noninferiority Trial
Source: Pain Res Manag. 2026 Apr 29;2026:7529711. doi: 10.1155/prm/7529711 (PMC13129405; doi:10.1155/prm/7529711)
Supplement: Supplementary file 1 — Supporting Information Additional supporting information can be found online in the Supporting Information section. [file PRM-2026-7529711-s001.docx]

**Supplementary Table 1 Eligibility Criteria**

| **Inclusion Criteria** | **Exclusion Criteria** |
| --- | --- |
| **Age**: 18–70 years (male or female)  **Surgery Type**:  Upper limb (clavicle/humerus fracture)  Lower limb (medial/lateral malleolus or calcaneal fracture)  Expected postoperative analgesia requirement ≥24h  **BMI**: 18–28 kg/m²  **ASA Physical Status**: I–III  **Cognitive/Functional Ability**:  Understands study procedures  Can operate PCA devices  Communicates effectively with researchers  **Informed Consent**: Voluntarily signs informed consent | **Liver Dysfunction**:  ALT or AST > 2×ULN  Total bilirubin ≥ 1.5×ULN  **Renal Impairment**:  Serum creatinine >112 μmol/L  Dialysis within 28 days preoperatively  **Bleeding Risk**:  Current anticoagulant/antiplatelet use  Congenital bleeding disorders (e.g., hemophilia)  Thrombocytopenia (PLT <50×10⁹/L) or platelet dysfunction (e.g., ITP, DIC)  Clinically significant active bleeding  **Cardiac Abnormalities**:  ECG abnormalities (investigator-assessed as ineligible)  Sick sinus syndrome or third-degree AV block (pacemaker-dependent)  Atrial fibrillation (ventricular rate >100 bpm) or frequent PVCs (>6/min)  **Uncontrolled Hypertension**:  Seated SBP ≥160 mmHg or DBP ≥105 mmHg despite treatment  **Uncontrolled Diabetes**: Fasting glucose ≥11 mmol/L  **Infection**:  Local infection at puncture site  Systemic severe infection  **Recent Trial Participation**: Other clinical trials within 30 days  **Neurological Conditions**:  Diabetic peripheral neuropathy  Other contraindications to nerve block  **Malignancy**: Active cancer  **Pregnancy/Lactation**: Confirmed by hCG test  **Other**: Investigator-determined ineligibility |

ULN, Upper limit of normal; BMI, Body mass index; ASA, American Society of Anesthesiologists; PCA, Patient-controlled analgesia; ALT/AST, Alanine/aspartate aminotransferase; TBIL, Total bilirubin; PLT, Platelet count; ITP, Idiopathic thrombocytopenic purpura; DIC, Disseminated intravascular coagulation; ECG, Electrocardiogram; AV, Atrioventricular; PVC, Premature ventricular contraction; SBP/DBP, Systolic/diastolic blood pressure; hCG, Human chorionic gonadotropin

**Supplementary Table 2 The 24-h postoperative analgesia effectiveness rate (Tuoren Group - Contiplex D Group) and 95% Confidence Intervals by Center, Unadjusted for Covariates**

|  | Rate difference（%） |  | 95% Confidence intervals (%) |  |
| --- | --- | --- | --- | --- |
|  |  | **Miettinen-Nurminen method** | **Newcombe-Wilson method** | **Newcombe-Wilson with continuity correction** |
| FAS |  |  |  |  |
| 1 | 0.00 | 0.00 | 0.00, 0.00 | 0.00, 0.00 |
| 2 | 3.03 | -7.42, 15.46 | -7.42, 15.32 | -9.93, 17.51 |
| 3 | -4.55 | -22.08, 11.60 | -21.80, 11.36 | -24.88, 15.17 |
| Total | 0.00 | -4.02, 4.02 | -3.99, 3.99 | -4.70, 4.70 |
| FAS (worst-case) |  |  |  |  |
| 1 | -1.69 | -7.49, 4.12 | -7.36, 3.99 | -8.74, 5.37 |
| 2 | 2.94 | -8.33, 15.49 | -8.33, 15.36 | -10.87, 17.56 |
| 3 | -4.55 | -22.08, 11.60 | -21.80, 11.36 | -24.88, 15.17 |
| Total | -0.88 | -5.04, 3.28 | -5.01, 3.25 | -5.73, 3.97 |
| PPS |  |  |  |  |
| 1 | 0.00 | 0.00, 0.00 | 0.00, 0.00 | 0.00, 0.00 |
| 2 | 3.03 | -8.01, 15.46 | -7.97, 15.32 | -10.62, 17.51 |
| 3 | 0.00 | 0.00, 0.00 | 0.00, 0.00 | 0.00, 0.00 |
| Total | 0.91 | -2.56, 4.99 | -2.61, 4.97 | -3.46, 5.69 |

△= adjusted rate difference; 95% CI is derived using the Miettinen-Nurminen method and *P* value is based on Wilcoxon rank sum test.

**Supplementary Table 3 The 24-h postoperative analgesia effectiveness rate (Tuoren Group - Contiplex D Group) adjusted for both types of surgery and center in PPS**

|  | Mantel-Haenszel method | |  | Stratified Newcombe (Mantel-Haenszel weighted) | |
| --- | --- | --- | --- | --- | --- |
| Covariate | Rate difference (%) | 95%CI |  | Rate difference (%) | 95%CI |
| Surgery | 0.87 | -0.87, 2.60 |  | 1.01 | -3.90, 5.92 |
| Center | 0.90 | -0.87, 2.68 |  | 0.73 | -4.59, 6.04 |
| Surgery and center | 0.90 | -0.87, 2.67 |  | 0.72 | -6.23, 7.67 |

△= adjusted rate difference; 95% CI is derived using the Miettinen-Nurminen method and *P* value is based on Wilcoxon rank sum test.

**Supplementary Table 4: Laboratory, electrocardiograms and chest X-rays Indicators for Treatment Period 48 Hours - Normal/Abnormal Status in Both Groups (SS)**

| **Item** | **Group** | **Treatment Period 48h Normal** | **Treatment Period 48h Abnormal** | **Pre-Screening Normal** | **Pre-Screening Abnormal** | **Missing Data** | **Total** |
| --- | --- | --- | --- | --- | --- | --- | --- |
| **Blood Routine** | **Hemoglobin** |  |  |  |  |  |  |
|  | *Tuoren* | 50 | 40 | 4 | 16 | 1 | 115 |
|  | *Contiplex D* | 57 | 34 | 0 | 22 | 0 | 114 |
|  | **Red Blood Cells** |  |  |  |  |  |  |
|  | *Tuoren* | 52 | 30 | 4 | 24 | 1 | 115 |
|  | *Contiplex D* | 47 | 37 | 4 | 25 | 0 | 114 |
|  | **Hematocrit** |  |  |  |  |  |  |
|  | *Tuoren* | 42 | 38 | 5 | 25 | 1 | 115 |
|  | *Contiplex D* | 44 | 39 | 1 | 28 | 0 | 114 |
|  | **Platelet Count** |  |  |  |  |  |  |
|  | *Tuoren* | 101 | 5 | 1 | 3 | 1 | 115 |
|  | *Contiplex D* | 103 | 2 | 3 | 5 | 0 | 114 |
|  | **hCG** |  |  |  |  |  |  |
|  | *Tuoren* | 0 | 0 | 0 | 0 | 12 | 103 |
|  | *Contiplex D* | 0 | 0 | 0 | 0 | 18 | 96 |
| **Coagulation** | **aPTT** |  |  |  |  |  |  |
|  | *Tuoren* | 103 | 3 | 2 | 2 | 0 | 115 |
|  | *Contiplex D* | 106 | 1 | 1 | 2 | 0 | 114 |
|  | **PT** |  |  |  |  |  |  |
|  | *Tuoren* | 107 | 1 | 0 | 0 | 7 | 115 |
|  | *Contiplex D* | 106 | 0 | 4 | 0 | 4 | 114 |
|  | **TT** |  |  |  |  |  |  |
|  | *Tuoren* | 109 | 1 | 0 | 0 | 5 | 115 |
|  | *Contiplex D* | 109 | 0 | 1 | 0 | 4 | 114 |
|  | **D-Dimer** |  |  |  |  |  |  |
|  | *Tuoren* | 10 | 19 | 6 | 65 | 10 | 115 |
|  | *Contiplex D* | 19 | 10 | 9 | 64 | 9 | 114 |
| **Blood Biochemistry** | **AST** |  |  |  |  |  |  |
|  | *Tuoren* | 87 | 11 | 11 | 2 | 0 | 115 |
|  | *Contiplex D* | 91 | 13 | 3 | 2 | 1 | 114 |
|  | **ALT** |  |  |  |  |  |  |
|  | *Tuoren* | 87 | 14 | 8 | 2 | 0 | 115 |
|  | *Contiplex D* | 93 | 10 | 5 | 1 | 1 | 114 |
|  | **Creatinine (sCr)** |  |  |  |  |  |  |
|  | *Tuoren* | 89 | 9 | 5 | 8 | 0 | 115 |
|  | *Contiplex D* | 90 | 3 | 6 | 10 | 1 | 114 |
|  | **BUN** |  |  |  |  |  |  |
|  | *Tuoren* | 92 | 9 | 9 | 0 | 1 | 115 |
|  | *Contiplex D* | 97 | 3 | 6 | 3 | 1 | 114 |
|  | **Serum K+** |  |  |  |  |  |  |
|  | *Tuoren* | 73 | 12 | 15 | 9 | 1 | 115 |
|  | *Contiplex D* | 69 | 14 | 21 | 4 | 0 | 114 |
|  | **Serum Na+** |  |  |  |  |  |  |
|  | *Tuoren* | 100 | 4 | 5 | 0 | 1 | 115 |
|  | *Contiplex D* | 101 | 1 | 5 | 1 | 0 | 114 |
|  | **Serum Cl-** |  |  |  |  |  |  |
|  | *Tuoren* | 106 | 0 | 3 | 0 | 1 | 115 |
|  | *Contiplex D* | 101 | 1 | 6 | 0 | 0 | 114 |
|  | **Glucose (GLU)** |  |  |  |  |  |  |
|  | *Tuoren* | 62 | 12 | 10 | 20 | 2 | 115 |
|  | *Contiplex D* | 73 | 8 | 12 | 10 | 3 | 114 |
| **ECG** | **ECG** |  |  |  |  |  |  |
|  | *Tuoren* | 39 | 21 | 12 | 29 | 2 | 12 |
|  | *Contiplex D* | 41 | 12 | 17 | 33 | 0 | 114 |
| **Chest X-ray** | **Chest X-ray** |  |  |  |  |  |  |
|  | *Tuoren* | 50 | 13 | 6 | 32 | 2 | 115 |
|  | *Contiplex D* | 51 | 11 | 10 | 33 | 0 | 114 |

**Laboratory indicators include**: Blood Routine (Hemoglobin, Red Blood Cells, Hematocrit, Platelet Count, Human Chorionic Gonadotropin), Coagulation Tests (aPTT, PT, TT, D-Dimer), Blood Biochemistry (ALT, AST, SCr, BUN, K+, Na+, Cl-, GLU), ECG, and Chest X-ray.

**Supplementary Table 5: Vital Signs During Different Visit Periods - Tuoren and Contiplex D groups**

|  | **Group** | **Normal at Screening Period** | | **Abnormal at Screening Period** | | **Missing Data** | **Total** |
| --- | --- | --- | --- | --- | --- | --- | --- |
|  |  | **Visit Period Normal** | **Visit Period Abnormal** | **Visit Period Normal** | **Visit Period Abnormal** |  |  |
| **Before Treatment (0h)** |  |  |  |  |  |  |  |
| **Systolic Blood Pressure** | Tuoren | 88 | 6 | 8 | 13 | 0 | 115 |
|  | Contiplex D | 92 | 11 | 1 | 10 | 0 | 114 |
| **Diastolic Blood Pressure** | Tuoren | 91 | 12 | 3 | 9 | 0 | 115 |
|  | Contiplex D | 103 | 4 | 1 | 6 | 0 | 114 |
| **Resting Heart Rate** | Tuoren | 110 | 1 | 4 | 0 | 0 | 115 |
|  | Contiplex D | 106 | 2 | 6 | 0 | 0 | 114 |
| **Oxygen Saturation** | Tuoren | 112 | 1 | 0 | 2 | 0 | 115 |
|  | Contiplex D | 112 | 1 | 0 | 1 | 0 | 114 |
| **Body Temperature** | Tuoren | 93 | 13 | 7 | 2 | 0 | 115 |
|  | Contiplex D | 96 | 9 | 4 | 5 | 0 | 114 |
| **During Treatment (24h)** |  |  |  |  |  |  |  |
| **Systolic Blood Pressure** | Tuoren | 89 | 4 | 20 | 1 | 1 | 115 |
|  | Contiplex D | 95 | 7 | 9 | 2 | 1 | 114 |
| **Diastolic Blood Pressure** | Tuoren | 98 | 4 | 12 | 0 | 1 | 115 |
|  | Contiplex D | 100 | 6 | 6 | 1 | 1 | 114 |
| **Resting Heart Rate** | Tuoren | 108 | 3 | 4 | 0 | 0 | 115 |
|  | Contiplex D | 106 | 2 | 6 | 0 | 0 | 114 |
| **Oxygen Saturation** | Tuoren | 111 | 0 | 2 | 0 | 2 | 115 |
|  | Contiplex D | 111 | 1 | 1 | 0 | 1 | 114 |
| **Body Temperature** | Tuoren | 82 | 24 | 6 | 3 | 0 | 115 |
|  | Contiplex D | 86 | 19 | 4 | 5 | 0 | 114 |
| **After Treatment (48h)** |  |  |  |  |  |  |  |
| **Systolic Blood Pressure** | Tuoren | 84 | 2 | 18 | 3 | 8 | 115 |
|  | Contiplex D | 92 | 4 | 8 | 3 | 7 | 114 |
| **Diastolic Blood Pressure** | Tuoren | 92 | 3 | 12 | 0 | 8 | 115 |
|  | Contiplex D | 96 | 4 | 5 | 2 | 7 | 114 |
| **Resting Heart Rate** | Tuoren | 107 | 3 | 4 | 0 | 1 | 115 |
|  | Contiplex D | 107 | 0 | 6 | 0 | 1 | 114 |
| **Oxygen Saturation** | Tuoren | 107 | 2 | 2 | 0 | 4 | 115 |
|  | Contiplex D | 111 | 0 | 1 | 0 | 2 | 114 |
| **Body Temperature** | Tuoren | 91 | 14 | 9 | 0 | 1 | 115 |
|  | Contiplex D | 91 | 13 | 5 | 4 | 1 | 114 |

**Normal Ranges** (from *Diagnostic Medicine*, edited by Wan Xuehong, 2013, 8th Edition, People's Medical Publishing House):

- **Systolic/Diastolic Blood Pressure:** [90-140)/[60-90) mmHg
- **Resting Heart Rate:** [60-100] beats/min
- **Oxygen Saturation:** [95-100]%
- **Body Temperature:** [35.9-37]℃


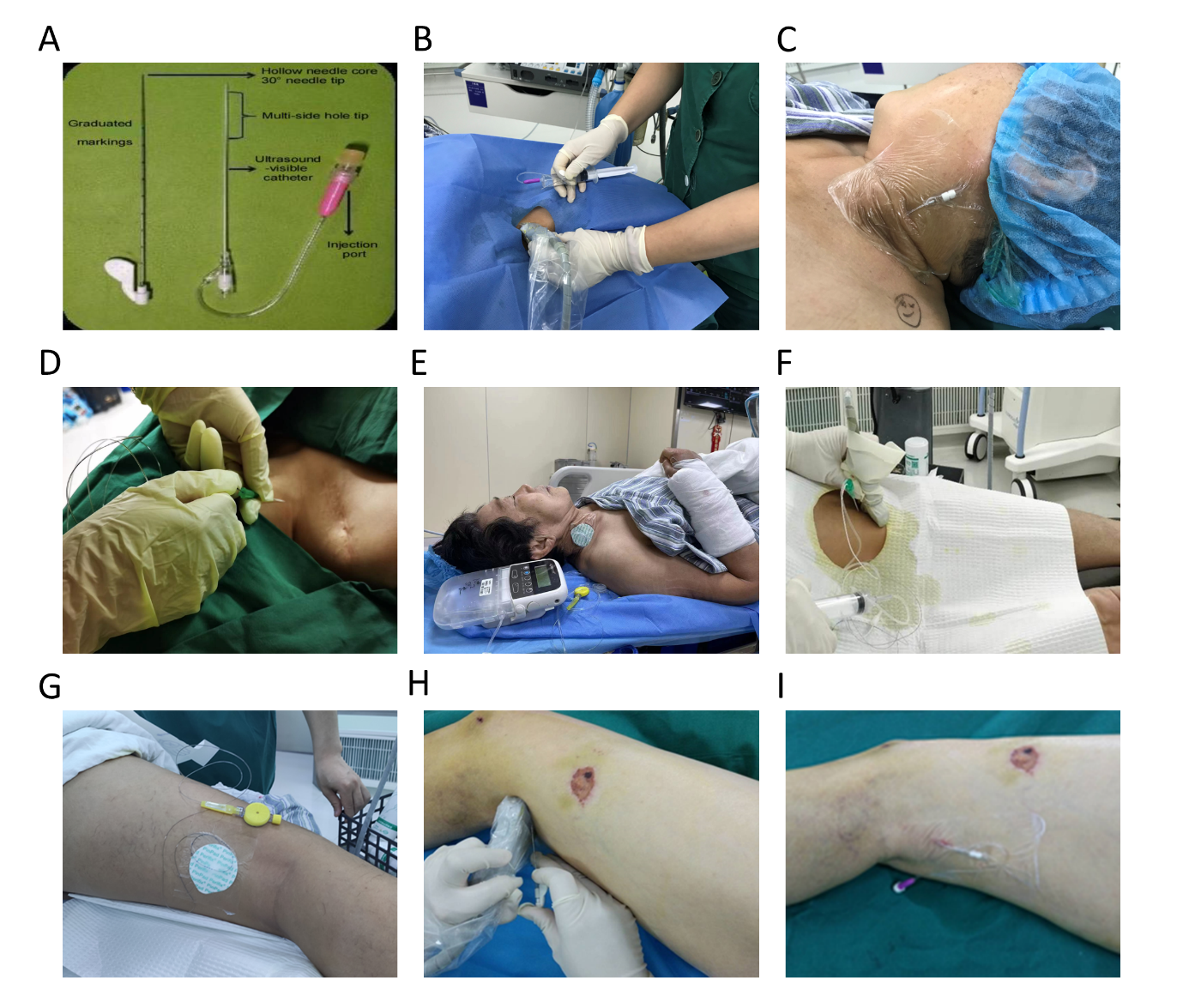


**Supplementary Figure 1. Diagram of patented Tuoren catheter-over-needle assembly applied to limbs facture surgery.** (A) Interior diagram of the patented package Tuoren catheter-over-needle assembly. The Tuoren catheter-over-needle assembly (B,C) or the Contiplex D catheter-through-needle assembly (D,E) applied in the upper limb continuous nerve block. The Tuoren catheter-over-needle assembly (F,G) or the Contiplex D catheter-through-needle assembly (H,I) applied in the lower limb continuous nerve block.
